# Heart-Focused Anxiety Affects Behavioral Cardiac Risk Factors and Quality of Life: A Follow-Up Study Using a Psycho-Cardiological Rehabilitation Concept

**DOI:** 10.3389/fpsyt.2022.836750

**Published:** 2022-05-09

**Authors:** Christoph Schmitz, Sonja Maria Wedegärtner, Eike Langheim, Judit Kleinschmidt, Volker Köllner

**Affiliations:** ^1^Psychosomatic Rehabilitation Research Group, Department of Psychosomatic Medicine, Center for Internal Medicine and Dermatology, Charité – Universitätsmedizin Berlin, Berlin, Germany; ^2^Department of Behavioral Psychotherapy, Technological University Dresden, Dresden, Germany; ^3^Department of Cardiology, Rehabilitation Center Seehof, Federal German Pension Agency, Teltow, Germany; ^4^Department of Behavioral Therapy and Psychosomatic Medicine, Rehabilitation Center Seehof, Federal German Pension Agency, Teltow, Germany

**Keywords:** psycho-cardiology, heart-focused anxiety, cardiac risk factors, health-related quality of life (HRQL), psychosomatic medicine, treatment outcome, rehabilitation, exercise capacity and physical activity

## Abstract

**Background:**

Heart-focused anxiety (HFA) raises the risk for adverse outcomes in patients with heart disease. Despite this great importance, it is rarely assessed in clinical practice. Three dimensions are commonly defined in the context of HFA: heart-related fear, avoidance, and attention. The impact of these aspects on cardiac risk factors is essentially unclear. In this study, we investigated the relationship between HFA and behavioral cardiac risk factors as well as health-related quality of life (HRQoL), which represent important treatment outcomes of inpatient psycho-cardiological rehabilitation.

**Methods:**

A prospective observational design was used to examine 238 rehabilitation inpatients with comorbidity of cardiac disease and psychiatric disorder. We assessed HFA using the Cardiac Anxiety Questionnaire (CAQ), HRQoL using the SF-12 Health Survey, exercise capacity using the 6-minute walk test, and smoking behavior, respectively at admission (t0) and discharge (t1). Physical activity was assessed at t0 and in a follow-up survey 6 months after discharge (t2) using the International Physical Activity Questionnaire (IPAQ). Multiple regression models were used to analyze the predictive value of HFA for the outcome variables at t0, t1, and t2, adjusted for socio-demographic factors and depression. Predictive values for changes over time were evaluated by the regressor variable approach.

**Results:**

Exercise capacity and physical activity were negatively predicted by baseline heart-related avoidance, both cross-sectionally and prospectively. Avoidance at t1 also negatively predicted long-term changes over time in physical activity at t2. Total HFA and the subcomponent avoidance negatively predicted physical HRQoL both cross-sectionally and prospectively. Mental HRQoL was cross-sectionally predicted by heart-focused attention at t0, and prospectively predicted by total HFA and by avoidance. Regarding changes in the course of rehabilitation, baseline avoidance negatively predicted improvement in physical HRQoL during rehabilitation. Concerning smoking behavior, no associations with HFA were found.

**Conclusions:**

HFA is a relevant inhibiting factor for the achievement of therapy goals in psycho-cardiological rehabilitation such as health behavior and HRQoL. Heart-related avoidance in particular, has a negative impact on exercise capacity, physical activity, and self-reported physical health. Its prospective negative predictive value for physical activity and physical health underlines the relevance of HFA for psycho-cardiological interventions.

## Introduction

Findings on the influence of psychological factors such as depression and anxiety on cardiac morbidity are frequently reported. While depression is a well-established independent risk factor for heart disease (1, 2), results regarding anxiety are inconsistent (3). Some studies report that anxiety is a risk factor for cardiac illness (1, 3–8), others do not confirm an independent association (9–11), and further results even point to protective effects of anxiety (12–14). Furthermore, adjusting for biobehavioral risk factors weakens the association between anxiety and cardiac morbidity (15, 16). In particular, smoking (17, 18), physical activity (18, 19), and exercise capacity (19–23) are known to be important prognostic factors regarding cardiac health. Although associations between behavioral cardiac risk factors and anxiety have been reported before (5, 24, 25), results are scarce and somewhat inconclusive (25, 26). Often no significant correlations were found (27, 28).

In view of these inconsistent findings, the concept of heart-focused anxiety (HFA) (29) can be useful to elucidate some of these relationships. HFA reflects a special pattern of anxiety symptoms with clinical relevance in cardiology, psychosomatic medicine, and especially in the field of psycho-cardiology (30). In differentiation to general anxiety, HFA comprises the fear of heart-related sensations, avoidance of triggering activities, and attention to heart-related symptoms (29). They represent cognitive-emotional, behavioral and cognitive-attentive aspects of HFA, respectively. Despite its relevance, HFA is rarely assessed in clinical practice.

While the concept of HFA was originally developed to capture noncardiac thoracic anxiety states (31), it is also relevant for patients suffering from heart disease. In a study by Van Beek et al. (32), HFA predicted the occurrence of further adverse cardiac events in patients with myocardial infarction. While their findings suggest this association to be driven mainly by avoidance of physical activity, unfortunately, no measures of physical activity were included in their data. Wedegärtner et al. reported that low quality of life and low physical activity predicted HFA in patients with heart failure (33). Bunz et al. (34) showed a high incidence of HFA in patients with heart failure, and an association with general anxiety, depression and quality of life. The results also suggest that the assessment of HFA in cardiac patients may add important information that is not captured by general anxiety alone. Limitations of the latter two studies arise from the cross-sectional analysis which allows no interpretation regarding the direction of the associations.

We followed a different approach, investigating the predictive value of HFA on behavioral cardiac risk factors and quality of life, including prospective analyses. This can contribute to a better understanding of the link between anxiety and adverse cardiac outcomes by emphasizing the behavioral pathway of a biobehavioral model of adverse outcomes in cardiac patients (35).

In the present study, we investigated the cross-sectional and prospective predictive value of HFA for treatment outcomes of psycho-cardiological rehabilitation. The inpatient treatment program was specifically developed to treat patients with comorbid psychosomatic and cardiological illness, aiming at improving cardiac prognosis and participation in life. In this context, we analyzed associations of HFA with smoking, exercise capacity, physical activity, and self-reported health-related quality of life (HRQoL). In the latter, the patient's perspective on health is presented, which is viewed as an important endpoint alongside clinical measures (36). In order to control for possible confounders, we included sociodemographic factors (SDF) and depressivity in our analyses.

To our knowledge, this is the first study investigating the impact of HFA on results of psycho-cardiological inpatient treatment regarding behavioral cardiac risk factors and HRQoL.

We hypothesize that the subdomains of heart-focused anxiety (fear, attention, and avoidance) differ in their predictive value for behavioral cardiac risk factors: As avoidance represents the behavioral aspect of HFA, we expected the association between this domain and the abovementioned behavioral factors to be primarily relevant, independently of depression and socio-demographic factors. Regarding smoking, our motive is mainly exploratory.

## Materials and Methods

### Data Acquisition and Design

Data were obtained as part of the research project “Effectivity of Psycho-Cardiological Rehabilitation (EvaPK),” which is registered in the International Clinical Trials Registry Platform under the ID DRKS00023370. All participants gave written informed consent, study information was provided according to the Declaration of Helsinki (37). Approval was granted by the ethical committee of the State Medical Board of Brandenburg, Germany, on Jan. 8^th^, 2019 (No. S1(a)/2019).

In a prospective observational design, we examined a sample of *N* = 238 psycho-cardiological rehabilitation inpatients before and after treatment. Our sample constituted the psychocardiological rehabilitation treatment arm of the EvaPK-Project. Subjects were recruited between April 2019 and January 2021 in a rehabilitation clinic of the German Pension Fund in Teltow, Germany. Inclusion criteria were age between 18 and 70 years, and presence of a somatic cadiac diagnosis that needs to be treated as well as a psychiatric diagnosis of the categories affective disorders, neurotic, stress-related and somatoform disorders or psychological and behavioral factors associated with disorders or diseases classified elsewhere. Exclusion criteria were acute psychotic or manic disorders, severe cognitive impairments or inability to comprehend diagnostic instructions or questionnaires. Pretreatment data were assessed during the week of admission (t0), posttreatment data were collected in the days before discharge (t1). A follow-up survey was conducted 6 months after discharge from the clinic (t2) in the form of mailed paper questionnaires.

### Clinical Setting and Intervention

All participants attended a psycho-cardiological rehabilitation program, which was specifically designed to address patients with comorbid psychosomatic disorders (e.g., depression, anxiety disorder, post-traumatic stress disorder) and cardiological diseases (e.g., coronary heart disease, arrhythmic disorders, structural heart defects) (38). The referral to the program was reviewed by independent physicians, ensuring that only patients with comorbid psycho-cardiological disorders were admitted. The program comprises elements of both cardiac (39) and psychosomatic rehabilitation (40), combining joint efforts of trained specialists in both disciplines. It includes interdisciplinary diagnostics and a focus on exercise therapy as well as concurrent psychotherapy (41). Psychotherapy included weekly 2 × 30 min of individual therapy and 2 × 90 min of a specific group therapy for psycho-cardiology patients, following a cognitive-behavioral therapy framework with focus on interventions to manage anxiety and depression and to improve coping. The movement therapy program consists of a basic program, endurance training, and additional selectable applications, e.g., respiratory therapy, yoga and Qi Gong. The basic program includes training of body awareness, muscular and balance training and mobilization (2 x 60 min/week). The endurance training is done 3 × weekly 30 min as controlled ergometer training and 2 × weekly walking. The training pulse was previously determined *via* an exercise ECG. Progressive muscle relaxation according to Jacobson is planned as a short (30 min in a sitting position) and a long form (45 min in a lying position). Attention control, muscle tone reduction, influencing pulse and blood pressure and transference in daily life are regularly addressed.

### Psychological Parameters

HFA was measured at t0 and t1using the Cardiac Anxiety Questionnaire (CAQ) (29) in its German version. The CAQ is the standard assessment instrument for heart-focused anxiety, developed by the research group around Georg Eifert, who contributed chiefly to the development of the concept (31). It contains 17 5-point Likert items, allowing calculation of a total sum score and scores for the three subscales fear, avoidance and attention, which are comprised of 8, 4 and 5 items, respectively. In a large validation study in a German general population, the postulated hierarchical factor structure of the sum score and the subscales fear, avoidance and attention was confirmed, and high internal consistencies of the scales were reported, ranging from Cronbach's α = 0.81 to α = 0.93 (42).

Perceived HRQoL was assessed *via* the SF-12 questionnaire (43) in its German version, a short form of the SF-36 health status questionnaire (44), which is widely used in a variety of clinical and research settings, and can be considered well validated (45). The SF-12 contains 12 items which yield a physical health component score and a mental health component score. These scales are computed following a 4-step algorithm (46), transformed to a range of possible values from 0 to 100, higher values corresponding to higher perceived quality of life. For standardization of the sum scores, the algorithm based on the German norm sample which was provided in the test manual (47) was used. HRQoL was assessed at t0 and t1.

As a possible confounder, depressivity was measured at t0 using the Revised Beck Depression Inventory (BDI-II) in its German version (48). The BDI-II is a self-report questionnaire allowing the computation of a total sum score by adding 21 four-point items that measure severity of depressive symptoms assessed in relation to the past 2 weeks. The BDI-II is widely used to measure depression symptom severity in clinical and research contexts and its psychometric properties can be considered good (49).

Psychiatric diagnoses were assessed using a standardized clinical interview, the Mini International Neuropsychiatric Interview (M.I.N.I.) in its German version (50). While being economical to use, the M.I.N.I. shows good psychometric properties and decent criterion validity (51).

### Behavioral Parameters

Smoking behavior was assessed *via* patients' self-reports regarding the number of cigarettes smoked at t0, and regarding smoking reduction or smoking cessation in the course of rehabilitation treatment at t1.

Exercise capacity was measured in 6-min walking distance (6MWD) tests. There is vast scientific evidence linking walking speed to cardiac morbidity (22, 23). The 6MWD is a performance test commonly used to assess exercise capacity and evaluate prognosis and treatment results in cardiovascular and respiratory diseases (52). It can be considered valid, reliable and sensitive to change (53). In the present study, 6MWD tests were conducted by trained hospital and study staff according to the standards suggested by the European Respiratory Society / American Thoracic Society (54). 6MWD tests were performed at t0 and t1.

Physical activity was quantified in MET-minutes per week in the International Physical Activity Questionnaire (IPAQ) self-report short form (55). The IPAQ was developed by an International Consensus Group aiming to assess standardized internationally comparable measures of physical activity on the population level. It shows acceptable measurement properties (56). Physical activity was assessed at t0 and in the follow-up survey at t2 *via* paper questionnaires. The IPAQ was not assessed at t1, because all patients received comparable amounts of physical exercise therapy during their rehabilitation treatment, thus interindividual differences in physical activity were expected to be rather small at t1. At t0, the completion of the questionnaires took place under supervision by study staff, that could provide help to participants. In order to avoid missing data in the follow-up at t2, we slightly modified the mailed IPAQ questionnaire by removing the check mark option for “Don't know/not sure” and adding a plea to fill in all data completely. In the cases of missing data in the questionnaires, no scores were computed.

### Cardiological Parameters

Cardiological diagnoses were assessed by specialist physicians on the basis of preliminary findings and clinical diagnostics, validated by senior physicians.

Patients rated their limitations *via* self-report according to the New York Heart Association (NYHA) Classification (57).

### Calculations

The software IBM SPSS^Ⓡ^ 27 was used for statistical analyses.

Initially, we calculated Pearson correlations between the CAQ scales at t0 and the continuous outcome variables at t0 and t1. Further, we calculated correlations between the CAQ scores at t1 and the IPAQ total score at t2. We also calculated correlations between all continuous variables and the potential confounders age and depressivity.

Multiple regression models were computed to analyze the predictive value of CAQ scores for the outcome variables smoking, exercise capacity, physical activity and HRQoL. Because relevant influences of SDF on HFA have been previously documented (42), we adjusted for the variables sex, age and education. We computed regression models for each of the outcome variables as criteria, using the CAQ total sum score and the subscores fear, avoidance and attention, respectively as predictors in separate models. In each case we employed hierarchical models, first entering the respective CAQ scale, in a second step adjusting for SDF, and in the third step entering the continuous BDI-II sum score as measure of depressivity in the models.

For analysis of cross-sectional associations, we computed regression analyses for all the baseline data at t0. Then, to analyze the prospective predictive value of HFA, we calculated regression models with CAQ scores as predictors, which preceded the outcome parameters as dependent variables in temporal order: First, we regressed the outcome variables at t1 on the baseline CAQ scores, respectively in separate models, adjusted for SDF and depressivity. Furthermore, to analyze prediction of change in the continuous outcome variables, we used a regressor variable approach (58), again regressing the outcome variables at t1 on the baseline CAQ scores, and additionally adjusting for the baseline-scores of the outcome variables.

In the case of physical activity, the IPAQ, as beforementioned, was not assessed at t1, but instead in the follow-up at t2. For analysis of the prospective predictive value of HFA on the follow-up results, we deemed the CAQ score at t1 of primary interest as a predictor, as it represents the result of therapeutic interventions and constitutes the most actual preceding value. Therefore, we regressed the IPAQ score at t2 as dependent variable on the CAQ scores at t1 as predictors.

For continuous outcome parameters (exercise capacity, physical activity, HRQoL), linear models were used. In the case of non-linearity and heteroskedasticity, we conducted bootstrap analyses with 2,000 samples, respectively, using bias corrected and accelerated (BCa) confidence intervals. For dichotomous outcome parameters (smoking status), logit models of the Generalized Linear Model were used. For count data (number of cigarettes smoked), we used Poisson loglinear models. In the case of overdispersion, we applied the chi-square scaling method for parameter estimation as suggested by McCullagh and Nelder (59).

Due to the small number of events in the case of smoking, we computed likelihood-penalized logistic regression analyses following the Firth method (60) in order to improve the quality of the model parameters. For this purpose, the STATS FIRTHLOG procedure of the R Essentials extension for SPSS was employed, which uses the R logistf package (61). For regression analyses with rare events, which was the case regarding smoking, we conducted *post-hoc* power analyses using G^*^Power Version 3.1 (62).

To compare participants with patients who declined participation and completers of the follow-up survey with non-completers, chi-square tests for nominal data and independent *t*-tests were used.

Generally, we considered results with *p* < 0.05 to be statistically significant in hypothesis testing. Regarding physical activity, exercise capacity and HRQoL, our hypotheses specifically concern the subscale avoidance. Regarding smoking, our research questions are exploratory and thus rather hypothesis-generating. Therefore, we did not deem adjustments for multiple testing necessary.

## Results

### Sample Characteristics

Two hundred eight Patients met the inclusion criteria, of which 238 gave written consent to participation and use of their data. The patients who declined participation did not significantly differ in age, sex or educational level. 130 (54.6%) male and 108 (45.4%) female patients with an age mean of 54.6 years (SD 7.48) participated in the study, 44 Patients (18.5%) reported smoking at admission (Table 1).

**Table 1:** Sample characteristics, NYHA classification and CAQ scores (*N* = 238).

		**M (SD)**	**Count**
Sex (count)	Male		130 (54.6%)
	Female		108 (45.4%)
Education level (count)	High		150 (63.0%)
	Low		88 (37.0%)
Smoking status (count)	No		186 (78.2%)
	Yes		44 (18.5%)
	No comment		8 (3.4%)
NYHA class	1		107 (45.1%)
	2		94 (39.7%)
	3		28 (11.8%)
	4		8 (3.4%)
Age (years)		54.63 (7.48)	
CAQ sum score		1.78 (0.64)	
CAQ fear		1.88 (0.77)	
CAQ avoidance		1.57 (1.00)	
CAQ attention		1.78 (0.67)	

*M, mean; SD, standard deviation; CAQ, Cardiac Anxiety Questionnaire; NYHA class, New York Heart Association classification system*.

All participants suffered from cardiovascular diseases and comorbid psychiatric diseases. Details about diagnoses are provided in Table 2. 45.1% of the participants fell into NYHA class I, 39.7% into NYHA class II, 11.8% into NYHA class III, and 3.4% into NYHA class IV (Table 2).

**Table 2:** Psychiatric and cardiac diagnoses in the sample (*N* = 238).

		**Frequency**	**Ratio**
Psychiatric diagnoses	Affective disorder	130	54.6%
	Anxiety disorder	119	50.0%
	Adjustment disorders	23	10.0%
	PTSD	26	10.9%
	Eating disorder	5	2.1%
	Hypochondria	9	3.8%
	Psychotic disorder	9	3.8%
	Alcoholism	7	2.9%
Cardiac diagnoses	Hypertension	74	31.1%
	Ischaemic heart diseases	115	48.3%
	Conduction/arrhythmic disorders	78	32.8%
	Structural defects, cardiomyopathy and heart failure	64	26.9%
	Cerebrovascular diseases	12	5.0%
	Other vascular diseases	15	6.3%
	Heart transplant or cardiac device implant	22	9.2%
	Other cardiac and circulatory diseases	15	6.3%

Sample means (and standard deviations in parentheses) of the CAQ scores at t0 were 1.78 (0.64) for the sum scale, 1.88 (0.77) for the subscale fear, 1.57 (1.00) for the subscale avoidance, and 1.78 (0.67) for the subscale attention (Table 1). Internal consistencies (Cronbach's alpha) were 0.87 for the CAQ sum scale, 0.81 for the subscale fear, and 0.90 for avoidance, which is high. The value 0.66 for attention can still be considered acceptable.

164 Patients (68.9%) completed the follow-up survey assessing physical activity *via* IPAQ six months after discharge from rehabilitation. 17 Patients sent back the questionnaires containing missing data, in which cases no scores could be calculated. The patients who sent back the questionnaires did not significantly differ at t0 in sex, educational status, HFA scores, BDI-II scores, IPAQ scores, 6MWD or physical HRQoL from patients who did not. They did show significantly lower age (mean difference = 2.37, T (236) = 2.29, *p* = 0.023) and lower mental HRQoL (mean difference = 4.38, T (224) = 2.69, *p* = 0.008).

### Cross-Sectional Associations Before Treatment

#### Correlations

The CAQ sum score showed highly significant correlations with SF-12 physical and mental health. The CAQ subscale fear showed a highly significant correlation with SF-12 mental health. The subscale avoidance was highly correlated with 6MWD, IPAQ score, SF-12 physical and mental health. The subscale attention showed highly significant correlations with SF-12 physical and mental health (Supplementary Table S1). There were no significant correlations between the CAQ scores and the number of cigarettes smoked prior to admission.

#### Regression Models

An overview of the results of cross-sectional regression analyses at t0 is presented in Table 3.

**Table 3:** Regression coefficients of the CAQ scales predicting health behaviors at baseline.

		**Target parameters (dependent variables)**					
		**Smoking amount**	**Smoking status**	**6MWD**	**IPAQ total MET-min/week**	**Physical health**	**Mental health**
	**Model**	***N* = 228**	***n* = 230**	***n* = 215**	***n* = 213**	***n* = 238**	***n* = 238**
CAQ total	Univariate	−0.26 (0.58)	Exp (B) = 0.71	−23.08^*^ (10.69)	−463.46 (352.84) (BCa)	−4.16^**^ (0.96)	−5.48^**^ (1.05)
		[−1.40, 0.88]	[0.43, 1.20]	[−44.14, −2.01]	[−1,144.36, 224.62] (BCa)	[−6.04, −2.27]	[−7.56, −3.41]
	Adjusted	−0.60 (0.61)	Exp (B) = 0.65	−11.47 (10.80)	−196.00 (262.19) (BCa)	−1.98^*^ (0.96)	−1.42 (0.81)
		[−1.79, 0.60]	[0.37, 1.13]	[−32.76, 9.82]	[−884.59.69, 481.18] (BCa)	[−3.88, −0.09]	[−3.02, 0.19]
CAQ fear	Univariate	−0.45 (0.48)	Exp (B) = 0.70	−3.58 (8.95)	220.33 (283.12) (BCa)	−1.07 (0.82)	−3.09^*^ (0.90)
		[−1.39, 0.50]	[0.45, 1.08]	[−21.21, 14.06]	[−361.08, 787.45] (BCa)	[−2.69, 0.55]	[−4.87, −1.32]
	Adjusted	−0.73 (0.48)	Exp (B) = 0.64	5.66 (8.60)	404.38 (286.44) (BCa)	0.43 (0.78)	−0.80 (0.65)
		[−1.68, 0.23]	[0.41, 1.01]	[−11.29, 22.61]	[−125.21, 938.37] (BCa)	[−1.10, 1.96]	[−2.09, −0.49]
CAQ avoidance	Univariate	0.49 (0.37)	Exp (B) = 1.08	−28.94^**^ (6.70)	−1,234.85^**^ (227.95) (BCa)	−4.34^**^ (0.56)	−3.32^**^ (0.67)
		[−0.29, 1.16]	[0.78, 1.50]	[−42.14, −15.74]	[−1,729.50, −804.94] (BCa)	[−5.45, −3.22]	[−4.65, −2.00]
	Adjusted	0.43 (0.39)	Exp (B) = 1.11	−27.17^**^ (6.85)	−1,182.82^**^ (244.54) (BCa)	−3.29^**^ (0.58)	−0.35 (0.52)
		[−0.33, 1.20]	[0.78, 1.59]	[−40.68, −13.66]	[−1,698.70, −741.84] (BCa)	[−4.43, −2.15]	[−0.68, 0.85]
CAQ attention	Univariate	−0.61 (0.55)	Exp (B) = 0.65	−13.27 (10.19)	298.15 (395.09) (BCa)	−2.74^*^ (0.92)	−4.36^**^ (1.01)
		[−1.69, 0.46]	[0.40, 1.06]	[−33.35, 6.82]	[−503.66, 1,091.16] (BCa)	[−4.55, −0.93]	[−6.35, −2.37]
	Adjusted	−0.90 (0.55)	Exp (B) = 0.59	−2.23 (9.89)	471.89 (387.45) (BCa)	−1.10 (0.88)	−1.74^*^ (0.73)
		[−1.99, 0.19]	[0.35, 1.00]	[−21.72, 17.26]	[−306.89, 1,230.78] (BCa)	[−2.83, 0.63]	[−3.19, −0.30]

*Univariate, univariate regression model with CAQ scale as predictor; adjusted, multiple regression model with CAQ scale, age, sex, educational status and depressivity as predictors*.

* *Statistical significance at the 0.05 level*;

** *Statistical significance at the 0.01 level. Standard errors are reported in parentheses; 95%-confidence intervals are reported in square brackets; (BCa) indicates bootstrap results (n = 2,000 samples). CAQ, Cardiac Anxiety Questionnaire; 6MWD, 6 min walking distance; IPAQ, International Physical Activity Questionnaire; “physical health” and “mental health” are subcomponents of the SF-12 Short Form Health Survey*.

##### Smoking Behavior

HFA was not found to be significantly associated with smoking behavior prior to admission. In logistic regression models, none of the CAQ scales turned out to be a statistically significant predictor of smoking status at t0. Power analyses revealed statistical powers of 63.3% for the CAQ sum scale, 67.7% for the subscale fear, and 12.0% for the subscale avoidance, which is not acceptable. The power of 80.3% for the subscale attention can be considered fair.

In Poisson loglinear regression models, none of the CAQ scales could predict the number of cigarettes smoked per day.

##### Exercise Capacity

We found the CAQ sum score to be a significant predictor of baseline 6MWD in the univariate model (F (1, 218) = 4.66, *p* = 0.032, adj. R^2^ = 0.02), but when adjusting for SDF, its coefficient lost the statistical significance. CAQ avoidance, however, showed a relevant predictive value (F (1,218) = 18.67, *p* < 0.001, adj. R^2^ = 0.08), which remained significant after adjustments for SDF and BDI-II sum score (95%-CI_coeff_[−40.68, −13.66], T = −3.96, *p* < 0.001). There was no significant predictive value of the CAQ subscales fear and attention on baseline 6MWD.

##### Physical Activity

For the regression models with the IPAQ total score before rehabilitation as dependent variable, we conducted bootstrapping analyses and used BCa confidence intervals due to skewedness of the data and heteroscedasticity.

We found the subscale avoidance to be a relevant negative predictor of physical activity (F (1, 211) = 26.47, *p* < 0.001, adj. R^2^ = 0.11) in the univariate model. Even after adjusting for SDF and depressivity, avoidance remained a relevant negative predictor with a statistically significant coefficient (BCa 95% CI_coeff_ [−1,698.70, −741.84], T = −4.84, *p* < 0.001). None of the other CAQ scores had significant predictive value for physical activity before rehabilitation.

##### Health-Related Quality of Life – Physical and Mental Health

Separate hierarchical multiple linear regression models were calculated for the two SF-12 components physical and mental health at t0 as dependent variables for each of the CAQ scales at t0 as predictors, respectively.

The CAQ sum score was a significant negative predictor of SF-12 physical health (F (1, 236) = 18.87, *p* < 0.001, adj. R^2^ = 0.07) and mental health scores (F (1, 236) = 27.13, *p* < 0.001, adj. R^2^ = 0.09) at t0 in univariate regression models. It remained a statistically significant negative predictor for physical health at t0 after adjusting for SDF and depressivity (95%- CI_coeff_ [−3.88, −0.89], T = −2.06, *p* = 0.040). Regarding mental health, the statistical significance for its coefficient was lost after adjustment for depressivity.

The CAQ subscore fear showed no significant predictive value for physical health at t0, but for mental health, its negative influence was statistically significant (F (1,236) = 11.82, *p* = 0.001, adj. R^2^ = 0.04) in a univariate regression model. After adjusting for depressivity, however, its coefficient lost the statistical significance.

The CAQ subscore avoidance showed relevant negative predictive value for physical health (F (1, 236) = 59.02, *p* < 0.001, adj. R^2^ = 0.20), and for mental health (F (1, 236) = 24.48, *p* < 0.001, adj. R^2^ = 0.09) at t0 in univariate models. Regarding physical health, its coefficient remained significant after adjustment for SDF and depressivity (95%- CI_coeff_[−4.43, −2.15], T = −5.69, *p* < 0.001). In regard to mental health, the influence was reduced well below the threshold of statistical significance after adjusting for depressivity.

The CAQ subscore attention turned out to be a negative predictor of physical health (F (1, 236) = 8.89, *p* = 0.003, adj. R^2^ = 0.03) and of mental health (F (1, 236) = 18.69, *p* < 0.001, adj. R^2^ = 0.07) at t0 in univariate models. Regarding physical health, the influence was reduced to be non-significant after adjusting for SDF and depressivity, but regarding mental health, its coefficient did remain statistically significant after adjustments (95%- CI_coeff_ [−3.19, −0.30], T = −2.37, *p* = 0.019).

### Prospective Predictive Value of CAQ Scores

An overview of the results of regression analyses regarding the predictive value of CAQ for the outcome parameters at t1 are presented in Table 4.

**Table 4:** Regression coefficients of the baseline CAQ scales predicting outcome parameters at discharge in separate regression models.

		**Target parameters at discharge (dependent variables)**				
		**6MWD**	**Physical health**	**Mental health**	**Smoking reduction**	**Smoking cessation**
	**Model**	***n* = 192**	***n* = 226**	***n* = 226**	***n* = 48**	***n* = 48**
CAQ total sum score	Univariate	−18.23 (12.03)	−3.84^**^ (1.02)	−5.42^**^ (1.16)	0.42 (0.48)	−0.81 (0.88)
		[−41.96, 5.51]	[−5.86, −1.82]	[−7.71, −3.12]	[−0.48, 1.38]	[−1.98, 0.22]
	Adjusted	3.94 (5.51)	−0.83 (0.81)	−1.34 (1.00)	-	-
		[−6.94, 14.82]	[−2.43, 0.77]	[−3.31, 0.64]		
CAQ fear	Univariate	0.01 (10.14)	−0.33 (0.65)	−2.70^**^ (0.99)	0.24 (0.39)	−0.78 (0.48)
		[−19.98, 20.00]	[−1.61, 0.95]	[−4.66, −0.74]	[−0.51, 1.02]	[−1.82, 0.10]
	Adjusted	1.81 (4.38)	−0.48 (0.63)	−0.36 (0.81)	-	-
		[−6.84, 10.47]	[−1.73, 0.76]	[−1.95, 1.23]		
CAQ avoidance	Univariate	−26.01^**^ (7.52)	−3.99^**^ (0.61)	−3.76^**^ (0.73)	0.22 (0.33)	−0.30 (0.36)
		[−40.83, −11.19]	[−5.20, −2.78]	[−5.20, −2.33]	[−0.40, 0.87]	[−1.03, 0.40]
	Adjusted	2.35 (3.64)	−1.09^*^ (0.54)	−1.21 (0.63)	-	-
		[−4.83, 9.54]	[−2.16, −0.02]	[−2.45, 0.04]		
CAQ attention	Univariate	−10.35 (11.43)	−2.16^*^ (0.98)	−4.05^**^ (1.11)	0.60 (0.52)	−0.55 (0.57)
		[−32.89, 12.19]	[−4.09, −0.23]	[−6.24, −1.86]	[−0.37, 1.65]	[−1.71, 0.54]
	Adjusted	3.50 (4.98)	−0.05 (0.74)	−1.02 (0.92)	-	-
		[−6.32, 13.33]	[−1.50, 1.40]	[−2.83, 0.79]		

*Univariate, model with respective CAQ scale as predictor; adjusted, model with respective CAQ scale, baseline IPAQ score, age, sex, educational status and depressivity as predictors. standard errors are reported in parentheses; 95%-confidence intervals are reported in square brackets*.

* *statistical significance at the 0.05 level*;

** *statistical significance at the 0.01 level. CAQ, Cardiac Anxiety Questionnaire; 6MWD, 6 min walking test; “physical health” and “mental health” are subcomponents of the SF-12 Short Form Health Survey*.

#### Correlations Between CAQ Scores at t0 and Outcome Variables at t1

The CAQ sum score showed highly significant correlations with SF-12 physical and mental health. The CAQ subscore fear showed a highly significant correlation with SF-12 mental health. Avoidance was highly correlated with 6MWD, SF-12 physical health and mental health. Attention showed a moderate correlation with SF-12 physical health and a highly significant correlation with SF-12 mental health (Supplementary Table S2).

#### Correlations Between CAQ Scores at t1 and Physical Activity at t2

Only CAQ avoidance at t1 significantly correlated with the physical activity IPAQ score at t2 (Supplementary Table S3).

#### Change in Smoking Behavior

At discharge from rehabilitation, 8 patients reported smoking cessation, 29 patients reported to have reduced the number of cigarettes per day, and 11 patients reported to smoke equally much as before treatment. None of the CAQ scores were statistically significant predictors for smoking cessation or for reducing the number of cigarettes smoked in logistic regression models. Regarding smoking cessation, we calculated statistical powers of 59.8% for the CAQ sum scale, 58.2% for the subscale fear, 19.1% for the subscale avoidance, and 38.7% for the subscale attention. Regarding smoking reduction, statistical powers of 32.0% for the CAQ sum scale, 16.9% for the subscale fear, 15.8% for the subscale avoidance, and 49.6% for the subscale attention were revealed. All these values cannot be considered acceptable.

#### Change in Exercise Capacity

Regarding the predictive value of CAQ for changes in exercise capacity in the course of rehabilitation treatment, we calculated hierarchical linear regression models with 6MWD at t1 as dependent variables. CAQ avoidance was a negative predictor of 6MWD at t1 in a univariate model [F (1,190) = 11.98, *p* = 0.001, adj. R^2^ = 0.05]. In a multivariate model including the baseline-6MWD score as predictor, avoidance did not significantly predict change in 6MWD. The CAQ sum score as well as the other CAQ subscores were not significantly associated with 6MWD at t1.

#### Change in Health-Related Quality of Life – Physical and Mental Health

Associations between CAQ and HRQoL at t1 were assessed by computing separate hierarchical linear regression models with the SF-12 subscales at t1 as dependent variables. The CAQ sum score showed a significant negative association with physical health [F (1,224) = 14.08, *p* < 0.001, adj. R^2^ = 0.06] and with mental health (F1,224) = 21.66, *p* < 0.001, adj. R^2^ = 0.08) at t1 in univariate regression models. After adjusting for baseline physical health, it showed no statistically significant predictive value for change in physical health. Regarding mental health, the CAQ sum score showed significant negative predictive value for improvement in mental health after adjusting for baseline mental health and for SDF (95%- CI_coeff_[−4.25, −0.16], T = −2.13, *p* = 0.034), but after adjusting for depressivity, its coefficient lost statistical significance.

The subscore avoidance at t0 did show a relevant negative association with physical health [F (1,224) = 42.58, *p* < 0.001, adj. R^2^ = 0.16] and with mental health [F (1,224) = 26.67, *p* < 0.001, adj. R^2^ = 0.10] at t1 in univariate regression models. After adjusting for baseline physical health, depressivity and SDF, it turned out to be an independent negative predictor for improvement in physical health (95%- CI_coeff_ [−2.16, −0.02], T = −2.00, *p* = 0.047). Regarding mental health, it remained statistically significant in negatively predicting improvement after adjusting for baseline mental health and for SDF (95%- CI_coeff_ [−3.20, −0.68], T = −3 .04, *p* = 0.003), but after adjusting for depressivity, its coefficient just slightly fell under the threshold of statistical significance (95%- CI_coeff_ [−2.45, 0.04], T = −1.91, *p* = 0.057).

CAQ fear at t0 showed no relevant predictive value for physical health at t1 in regression models, but in a univariate model, it did show predictive value for mental health at t1 (F (1,224) = 7.35, *p* = 0.007, adj. R^2^ = 0.03). However, when adjusting for baseline mental health, fear revealed no statistically significant predictive value for change in mental health.

The subscore attention at t0 did show a relevant negative association with physical health [F (1,224) = 4.86, *p* = 0.029, adj. R^2^ = 0.02] and with mental health [F (1,224) = 13.28, *p* < 0.001, adj. R^2^ = 0.05] at t1 in univariate regression models. The predictive value of attention for change in physical health as well as for change in mental health was reduced below statistical significance after adjustment for the respective baseline values, though.

#### Long-Term Change in Physical Activity

The association of CAQ scores with long-term changes in physical activity measured 6 months after discharge from rehabilitation was assessed in hierarchical linear regression models with IPAQ total MET-minutes per week at t2 as dependent variable. Due to skewedness of distribution and heteroskedasticity, bootstrapping was applied.

Table 5 shows an overview of the regression results regarding the follow-up data at t2.

**Table 5:** Regression coefficients of the CAQ scores at discharge predicting physical activity at follow-up (*N* = 144) in separate regression models.

	**Outcome (dependent variable)**	
	**IPAQ (MET-min/week) at follow up**	
	**Univariate**	**Adjusted**
CAQ sum score	−410.69 (348.40)	−267.40 (428.34)
	[−1106.04, 293.95]	[−1088.94, 579.45]
CAQ fear	−138.23 (375.34)	−137.40 (389.48)
	[−880.20, 603.74]	[−907.56, 632.77]
CAQ avoidance	−839.46 (231.17)^**^	−609.30 (242.93)^*^
	[−1292.33, −384.79]	[−1055.96, −166.96]
CAQ attention	363.25 (371.86)	410.24 (413.59)
	[−316.95, 1040.99]	[−339.38, 1188.65]

*Univariate, univariate regression model with respective CAQ scale as predictor: adjusted = multiple regression model with CAQ scale as predictor, adjusted for baseline IPAQ score and for age, sex, educational status and depressivity. Standard errors are reported in parentheses; 95%-confidence intervals are reported in square brackets. All standard errors and confindence intervals are results of bootstrap analyses with n = 2,000 samples*.

* *Statistical significance at the 0.05 level*;

** *Statistical significance at the 0.01 level. CAQ, Cardiac Anxiety Questionnaire; IPAQ, International Physical Activity Questionnaire*.

We found CAQ avoidance at t1 to be a significant negative predictor of physical activity at t2 (F (1,142) = 9.012, *p* = 0.003, adj. R^2^ = 0.05), also after adjustment for confounders. After adjusting for the baseline-IPAQ score, avoidance at t1 proved to be a significant negative predictor of change in physical activity (BCa 95% CI_coeff_ [−1,055.96, −166.96], T = −2.51, *p* = 0.011), independently of SDF and depressivity. No significant associations between the other CAQ scores and physical activity at t2 were found.

## Discussion

The aim of this study was to investigate the impact of heart-focused anxiety on behavioral cardiac risk factors and HRQoL, constituting relevant treatment outcomes of psycho-cardiological rehabilitation. Our results suggest that heart-focused anxiety is a relevant inhibiting factor for achieving therapy goals in psycho-cardiological rehabilitation. Avoidance, in particular, has a negative impact on exercise capacity, physical activity, and self-reported quality of life, both cross-sectionally and prospectively. Avoidance also negatively affects outcomes of psycho-cardiological inpatient treatment regarding physical HRQoL as well as long-term improvements in physical activity.

### Psychopathology and HFA in the Sample

HFA scores in our sample of psycho-cardiological rehabilitation inpatients are comparable with previously reported values assessed in cardiology patients before surgery (63). In comparison to non-cardiological or cardiological rehabilitation inpatient samples (63–65) and patients with cardiac arrythmia or with panic disorder (64), HFA levels in our sample are high. This is understandable, as the indication for psycho-cardiological rehabilitation encompasses problems like anxiety in combination with cardiac disease. As also 50.1% of the patients in our sample were diagnosed with anxiety disorder, we can conclude that our findings regarding HFA can be understood to some extent by psychopathological processes rather than normal psychological dynamics.

### Outcome Parameters

#### Smoking

18.5% of the patients in our sample reported smoking, which is low compared to the estimated population prevalence of 24.3% for adults over 15 years in Germany (66). Of the smokers, 16.7% self-reportedly quit smoking during rehabilitation, and 60.4% stated to have reduced consumption.

HFA was not associated with smoking behavior, neither cross-sectionally nor prospectively. Here, our results diverge from previous findings that link anxiety to smoking behavior (32, 67, 68). Possible explanations could be either obscuring effects of frequent comorbidities in our sample. Also, it is possible that quite some patients might have already successfully quitted smoking prior to admission to rehabilitation, thus leaving only the most committed smokers in the sample. This matches the relatively low rate of smokers in our sample. However, it is important to note that due to the low statistical powers (i.e., high type two errors), these results cannot be properly interpreted, as the small sample may have prevented the detection of actual associations in the population.

#### Exercise Capacity

Heart-related avoidance negatively predicted exercise capacity as measured by 6MWD at baseline as well as at the end of treatment, independently of SDF and depression, with small effect sizes (adj. R^2^ = 0.08 and 0.05, respectively). To our knowledge, this is the first study that examined this relationship. However, exercise capacity has been suggested before to be associated with general anxiety (69, 70). Exercise capacity is a vital measure for risk stratification and prognosis in patients with cardiac diseases (22, 23, 71). Given a sensitivity of psychological constraints to psychological intervention, targeting heart-focused anxiety could improve outcome in these patients.

#### Physical Activity

Avoidance negatively predicted physical activity at baseline and follow-up 6 months after rehabilitation, independently of SDF and depression, with small to medium effect sizes (adj. R^2^ = 11 and 5%, respectively). Moreover, avoidance at the end of treatment even negatively predicted improvement in physical activity assessed 6 months after rehabilitation treatment. Especially this prospective association underlines the relevance of avoidance in the psycho-cardiological scope. To our knowledge, this is the first work that shows an association between physical activity and HFA in patients under psycho-cardiological rehabilitation treatment, and the results match previous findings that show negative association between physical activity and HFA, especially avoidance (33, 72).

Engaging in regular moderate physical activity is essential in secondary prevention of cardiovascular diseases (19, 73, 74), and it can improve not only functional capacity, but also psychological factors as depression, anxiety and quality of life in cardiac patients (75–77). It may even have reverse effects on HFA, as a study with a sample recruited from emergency room visitors revealed protective effects of regular physical activity on noncardiac chest pain (78). Thus, identifying avoidance as a relevant psychobehavioral influencing factor may point to further specific interventions helping to improve psychopathologic and cardiac prognosis.

#### Health-Related Quality of Life

Total HFA and the subscale avoidance negatively predicted physical HRQoL at baseline (adj. R^2^ = 0.07 or total HFA and 0.20 for avoidance) as well as prospectively at the end of treatment (adj. R^2^ = 0.06 for total HFA and 0.16 for avoidance) after adjustments for SDF and depression. Mental HRQoL at the end of treatment was prospectively predicted by total HFA (adj. R^2^ = 0.08) and by avoidance (adj. R^2^ = 0.10), independently of SDF and depression. Regarding changes in HRQoL in the course of rehabilitation, baseline avoidance negatively predicted improvement in physical HRQoL during treatment, independently of SDF and depression.

HRQoL, in contrast to the behavioral outcome variables, shows not only independent associations with avoidance, but also with total HFA. This can be theoretically explained by the psychological dimension of HRQoL, which is also represented in the other components of HFA, fear and attention. Our results are in line with previous findings showing associations between HFA and reduced quality of life in cardiac patients (33, 34, 79, 80). Especially avoidance and fear have previously been identified as independent predictors of physical HRQoL (81). Our findings regarding HRQoL are of great relevance, as HRQoL represents an important outcome measure for clinical interventions and constitutes a central public health goal (82).

### Synopsis and Implications

Our results confirm the hypothesis that heart-related avoidance is associated with behavioral cardiac risk factors and quality of life. It negatively predicted exercise capacity, physical activity and physical HRQoL. These associations were evident both cross-sectionally and prospectively. Our results suggest that avoidance even inhibits therapeutic success of psycho-cardiological inpatient treatment regarding physical HRQoL as well as long-term improvements in physical activity.

This is in accordance with previous findings indicating avoidance to be the foremost important component of HFA in the prediction of cardiac morbidity (32), and suggesting prospective negative effects of anxiety on various treatment outcomes (69).

Our findings can contribute to explain the negative impact of anxiety on cardiac outcomes. The special concept of HFA can provide more differentiated contribution to the previously inconsistent research findings. There are several theories outlining biophysiological mechanisms that might explain this adverse association, including autonomic dysfunction and electrical instability. Also, HFA may negatively affect therapy adherence, as could be demonstrated for general anxiety in previous findings (83). Our results, on the other hand, emphasize the important role of avoidance in the behavioral pathway of a biobehavioral model (35). They suggest that regarding behavioral pathways, not only unhealthy coping styles, but also specifically avoidance of exercise may be a relevant factor underlying the association between anxiety and cardiac morbidity. This can open up opportunities for more specific interventions to further improve cardiac prognosis and HRQoL.

### Psycho-Cardiological Interventions to Improve Avoidance

Many patients with HFA avoid physical activity because the body sensations it induces (faster and stronger heartbeat, slight dyspnea) trigger anxiety. In a pure talking therapy, HFA related avoidance often cannot be overcome. Interdisciplinary psycho-cardiological interventions, especially the close cooperation of behavioral psychotherapy and exercise therapy, can offer great possibilities to specifically address this problem. Here, exercise therapy can take over the role of exposure *in vivo*, which is one of the most effective interventions in the therapy of anxiety (84). An experienced exercise therapist is able to recognize even subtle forms of avoidance behavior. In a psycho-cardiological rehabilitation setting, the exercise therapist reports this back to the patient as well as to the physician and psychotherapist, thus providing impulses for further psychotherapeutic processes. Additionally, the increase in fitness that can be achieved by exercise therapy in the course of rehabilitation, can correct dysfunctional beliefs about exertion being harmful, and can even establish optimism regarding potential for improvement of the physical condition.

## Limitations

As this is an observational study, assumptions pointing to causality are difficult. However, the prospective approach in our statistical analysis does apply causality's prerequisite of temporal order, which allows assertions regarding the direction of the associations.

Regarding the follow-up survey, selection bias is possible, especially since we found differences in age and mental HRQoL beween completers and dropouts. As our focus was on associations, however, we are not apprehensive of major effects arising from these differences.

Further, no objective measures of cardiac illness severity or functional impairments were included in our analysis. Thus, mediating effects are possible. For example, it seems plausible that higher avoidance may correspond with more severe physical impairment, which in turn can negatively affect physical activity, exercise capacity and HRQoL. In this matter, the capacity of the CAQ to differentiate between anxiety-related avoidance on one hand, and avoidance elicited by physical impairment on the other, is questionable. Nevertheless, several studies have shown that associations between anxiety and cardiac illness severity are not proportional. Several studies report no or weak associations of general anxiety with measures of cardiac disease severity (10, 85–87), but rather with subjective symptom perception. Specifically regarding HFA, it has repeatedly been found that cardiac anxiety could not be predicted by measures of cardiac disease severity (34, 65). O'Donovan et al. found that illness perception was a better predictor of HFA than illness severity (88). Moreover, even inverse relations between HFA and severity of cardiac illness have been reported (33, 89). Also, Van Beek et al. (32) found a prognostic effect of HFA on adverse cardiac outcomes independent of disease severity. Thus, there is support by previous research results that the impact of avoidance on behavioral risk factors and HRQoL can be at least partially accounted for by psychological and behavioral factors.

### Future Directions of Research

In the light of the predictive value of heart-related avoidance, further research should more precisely elucidate associations with behavioral risk factors, HRQoL and cardiac morbidity. Employing more sophisticated longitudinal designs or stepwise treatment blocks, controlling for relevant biological factors and especially objective measures of cardiac disease severity may be adequate to gain more etiological understanding. Concerning smoking, larger samples are required to investigate associations with HFA.

In addition, targeting HFA and, in particular, avoidance, could improve outcome parameters in patients undergoing psycho-cardiological rehabilitation and should also be subject of further research.

### Conclusions

Heart-focused anxiety, especially heart-related avoidance, is a predictor for reduced exercise capacity and a less physically active lifestyle before rehabilitation and in the long-term course afterwards. It should be regularly assessed and addressed in treatment, as it negatively impacts health behavior and quality of life.

## Data Availability Statement

The raw data supporting the conclusions of this article will be made available by the authors, without undue reservation.

## Ethics Statement

The study involving human participants was reviewed and approved by the State Medical Board of Brandenburg, Germany, on January 8th, 2019 (No. S1(a)/2019). The patients/participants provided their written informed consent to participate in this study.

## Author Contributions

CS was responsible for conceptualization of the study, data acquisition, data analysis, and writing and editing of the original manuscript. SW provided support in the preparation and formulation of the manuscript with relevant contributions to the introduction, results section, and discussion. EL developed the cardiology part of the intervention and contributed the cardiology parts of the manuscript. JK developed the exercise therapy part of the intervention and wrote the relevant part of the manuscript. Data were provided by the research project Effectivity of Psycho-Cardiological Rehabilitation (EvaPK), VK being the principal researcher and CS being the project coordinator. VK developed the study design, supervised the project and the writing of the manuscript, and assisted CS with the writing. All authors contributed to the article and approved the submitted version.

## Funding

This research project EvaPK, which provided the data for this study, was funded by a research grant from the Federal German Pension Agency (Grant No. 8011 - 106 - 31/31.127.1).

## Conflict of Interest

The authors declare that the research was conducted in the absence of any commercial or financial relationships that could be construed as a potential conflict of interest.

## Publisher's Note

All claims expressed in this article are solely those of the authors and do not necessarily represent those of their affiliated organizations, or those of the publisher, the editors and the reviewers. Any product that may be evaluated in this article, or claim that may be made by its manufacturer, is not guaranteed or endorsed by the publisher.
