# Supplementary material for: Heart-Focused Anxiety Affects Behavioral Cardiac Risk Factors and Quality of Life: A Follow-Up Study Using a Psycho-Cardiological Rehabilitation Concept
Source: Front Psychiatry. 2022 May 9;13:836750. doi: 10.3389/fpsyt.2022.836750 (PMC9124936; doi:10.3389/fpsyt.2022.836750)
Supplement: Supplementary file 1 [file Table_1.pdf]

Table S1. Pearson correlations with two-tailed significance at baseline

	CAQ fear	CAQ avoidance	CAQ attention	BDI-II	age	smoking	6MWD	IPAQ total	physical health	mental health
CAQ sum score	**0.898**<br>p<.001	**0.678**<br>p<.001	**0.772**<br>p<.001	**0.359**<br>p<.001	0.033<br>p=.617	-0.030<br>p=.655	-0.145<br>p=.032	-0.080<br>p=.247	**-0.272**<br>p<.001	**-0.321**<br>p<.001
CAQ fear	.	**0.379**<br>p<.001	**0.618**<br>p<.001	**0.245**<br>p<.001	0.017<br>p=.797	-0.062<br>p=.350	-0.027<br>p=.690	0.046<br>p=.502	-0.084<br>p=.195	**-0.218**<br>p=.001
CAQ avoidance		.	**0.296**<br>p<.001	**0.386**<br>p<.001	0.032<br>p=.624	0.078<br>p=.243	**-0.281**<br>p<.001	**-0.334**<br>p<.001	**-0.447**<br>p<.001	**-0.307**<br>p<.001
CAQ attention			.	**0.253**<br>p<.001	0.037<br>p=.572	-0.075<br>p=.261	-0.088<br>p=.194	0.055<br>p=.427	**-0.191**<br>p=.003	**-0.271**<br>p<.001
BDI-II				.	0.012<br>p=.855	0.036<br>p=.589	-0.138<br>p=.041	-0.166<br>p=.015	**-0.401**<br>p<.001	**-0.727**<br>p<.001
age					.	-0.101<br>p=.127	-0.136<br>p=.044	0.106<br>p=.124	-0.143<br>p=.027	0.050<br>p=.438
smoking						.	-0.016<br>p=.817	0.001<br>p=.986	-0.066<br>p=.323	-0.004<br>p=.950
6MWD							.	0.037<br>p=.601	**0.496**<br>p<.001	0.075<br>p=.265
IPAQ total								.	0.056<br>p=.410	0.127<br>p=.064
physical health									.	**0.181**<br>p=.005

Correlations in bold type are significant at the 0.01 level (2-tailed). CAQ = Cardiac Anxiety Questionnaire; BDI-II = Revised Beck Depression Inventory; 6MWD = 6-minute walking distance; 'smoking' measured number of cigarettes smoked per day; IPAQ = International Physical Activity Questionnaire (MET-minutes/week); 'physical health' and 'mental health' are subcomponents of the SF-12 Short Form Health Survey
