# Supplementary material for: Heart-Focused Anxiety Affects Behavioral Cardiac Risk Factors and Quality of Life: A Follow-Up Study Using a Psycho-Cardiological Rehabilitation Concept
Source: Front Psychiatry. 2022 May 9;13:836750. doi: 10.3389/fpsyt.2022.836750 (PMC9124936; doi:10.3389/fpsyt.2022.836750)
Supplement: Supplementary file 2 [file Table_2.pdf]

Table S2. Pearson correlations with two-tailed significance: Baseline CAQ and outcome parameters after treatment

	CAQ fear t0	CAQ avoidance t0	CAQ attention t0	BDI-II t0	age	6MWD t1	physical health t1	mental health t1
CAQ sum score t0	**0.898**<br>p<.001	**0.678**<br>p<.001	**0.772**<br>p<.001	**0.359**<br>p<.001	0.033<br>p=.617	-0.116<br>p=.099	**-0.243**<br>p<.001	**-0.297**<br>p<.001
CAQ fear t0	.	**0.379**<br>p<.001	**0.618**<br>p<.001	**0.245**<br>p<.001	0.017<br>p=.797	-0.007<br>p=.923	-0.086<br>p=.198	**-0.178**<br>p=.007
CAQ avoidance t0		.	**0.296**<br>p<.001	**0.386**<br>p<.001	0.032<br>p=.624	**-0.240**<br>p=.001	**-0.399**<br>p<.001	**-0.326**<br>p<.001
CAQ attention t0			.	**0.253**<br>p<.001	0.037<br>p=.572	-0.075<br>0.286	-0.146<br>0.029	**-0.237**<br>p<.001
BDI-II t0				.	0.012<br>p=.855	-0.166<br>0.018	**-0.315**<br>p<.001	**-0.616**<br>p<.001
age					.	**-0.189**<br>p=.007	-0.155<br>p=.019	0.018<br>p=.783
6MWD t1						.	**0.538**<br>p<.001	**0.291**<br>p<.001
physical health t1							.	**0.378**<br>p<.001

Correlations in bold type are significant at the 0.01 level (2-tailed). t0 = baseline; t1 = after treatment; CAQ = Cardiac Anxiety Questionnaire; BDI-II = Revised Beck Depression Inventory sum score; 6MWD = 6-minute walking distance; 'physical health' and 'mental health' are subcomponents of the SF-12 Short Form Health Survey.
