# Supplementary material for: Heart-Focused Anxiety Affects Behavioral Cardiac Risk Factors and Quality of Life: A Follow-Up Study Using a Psycho-Cardiological Rehabilitation Concept
Source: Front Psychiatry. 2022 May 9;13:836750. doi: 10.3389/fpsyt.2022.836750 (PMC9124936; doi:10.3389/fpsyt.2022.836750)
Supplement: Supplementary file 3 [file Table_3.pdf]

Table S3. Correlations and two-tailed significance: CAQ at discharge and physical activity at follow-up

	CAQ sum score t1	CAQ fear t1	CAQ avoidance t1	CAQ attention t1	age	BDI-II t0
IPAQ (MET-min/wk) t2	-0.062<br>p=.442	-0.007<br>p=.930	**-0.240**<br>p=.003	0.084<br>p=.297	0.048<br>0.538	-0.068<br>p=.385
BDI-II t0	**0.368**<br>p<.001	**0.285**<br>p<.001	**0.321**<br>p<.001	**0.322**<br>p<.001	0.012<br>p=.855	
age	0.086<br>p=.196	0.065<br>p=.331	0.074<br>p=.264	0.082<br>p=.219		

Correlations in bold type are significant at the 0.01 level (2-tailed). t0 = baseline; t1 = discharge; t2 = 6 months follow-up; CAQ = Cardiac Anxiety Questionnaire; BDI-II = Revised Beck Depression Inventory sum score; IPAQ = International Physical Activity Questionnaire.
